# Supplementary material for: STING induces ZBP1-mediated necroptosis independently of TNFR1 and FADD
Source: Nature. 2025 Aug 20;647(8090):735–46. doi: 10.1038/s41586-025-09536-4 (PMC12629989; doi:10.1038/s41586-025-09536-4)
Supplement: Supplementary file 1 — Supplementary Figs. 1–9 and Supplementary Tables 1–9. [file 41586_2025_9536_MOESM1_ESM.pdf]

nature portfolio

https://doi.org/10.1038/s41586-025-09536-4

Supplementary information

STING induces ZBP1-mediated necroptosis independently of TNFR1 and FADD

In the format provided by the authors and unedited

Supplementary Figure 1

Figure 1d

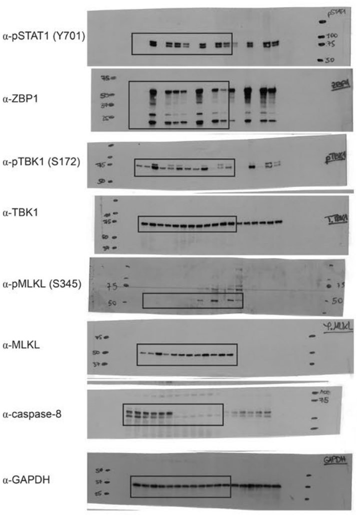

Figure 1e

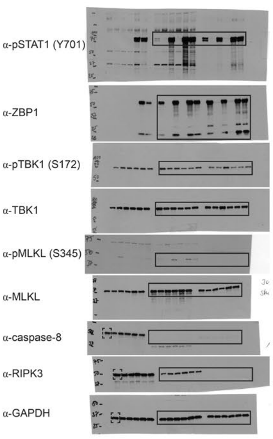

Figure 1f

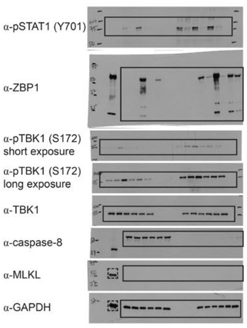

Figure 1g

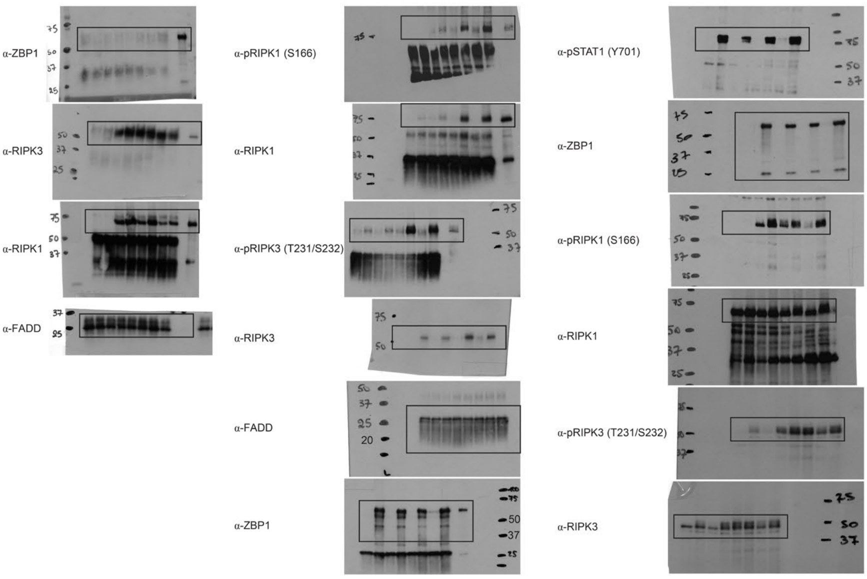

Figure 1h

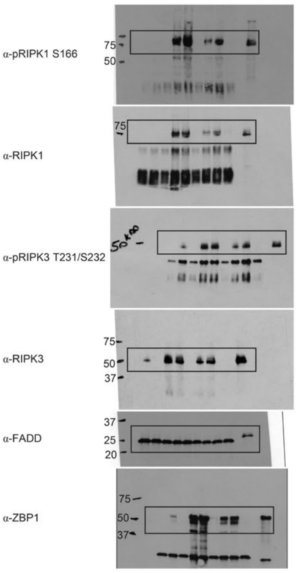

Figure 1h (continue)

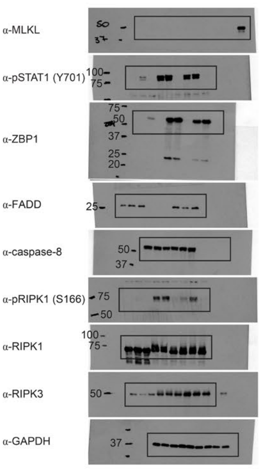

Figure 1i

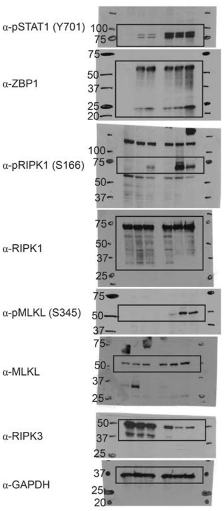

Extended Data Figure 2a

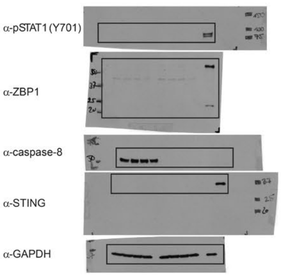

Extended Data Figure 6e

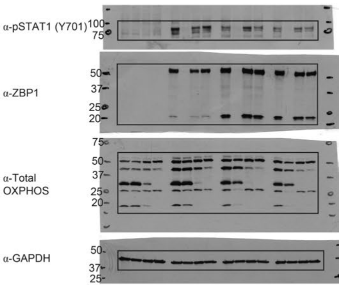

Uncropped western blot images. GAPDH was used as a loading control and was blotted on the same membranes, provided that no proteins of interest ran near ~37 kDa. At least two membranes per western blot/figure were used to ensure proper loading control assessment.

Supplementary Figure 2

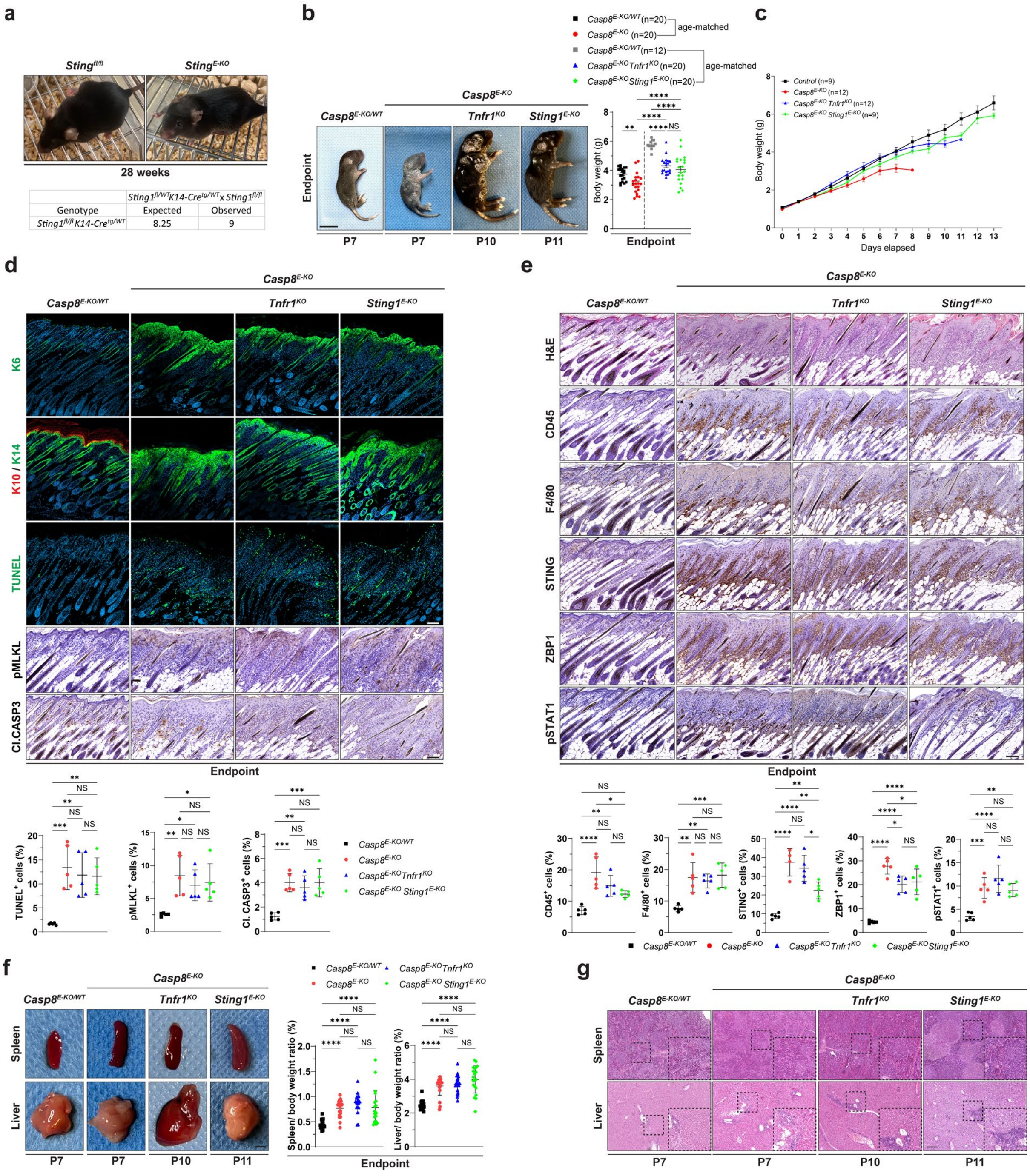

a, Representative images of *Sting1*$^{E\text{-}KO}$ and *Sting1*$^{fl/fl}$ mice and table of expected and observed numbers of weaned offspring with the designated genotypes resulting from intercrosses of parents with the indicated genotypes. **b**, Representative images and graphs of body weight of *n* mice with the indicated genotypes at their survival endpoint as indicated. Scale bar: 1 cm. **c**, Graph of the trajectory of body weight changes in mice with the indicated genotypes. *n* values are indicated. **d**, **e**, Representative images of consecutive skin sections from mice with the indicated genotypes stained for K6 (Alexa Fluor 488, green), K10 (Alexa Fluor 594, red),

K14 (Alexa Fluor 488, green), TUNEL (fluorescein, green), nuclei (DAPI, blue), pMLKL-S345, Cl.CASP3 (**d**) and H&E, CD45, F4/80, STING, pSTAT1-Y701 and ZBP1 (**e**) at the indicated survival endpoint. *n*=5 per group. Scale bars: 100 µm. Graphs show immunostaining quantification. **f**, **g**, Representative images, graphs of organ-to-body-weight ratio (**f**) and representative images of tissue sections stained with H&E (**g**) of spleen and liver of mice with the indicated genotypes at survival endpoint as indicated. *n*=20 per group. Control mice included age-matched *Casp8$^{E\text{-}KO/WT}$* littermates. Scale bars: 2 mm (spleen and liver images), 200 µm (bright-field images) and 50 µm (inserts). In **b**-**f**, data are presented as mean ± s.e.m.; each dot represents one mouse; control mice included age-matched *Casp8$^{E\text{-}KO/WT}$* littermates; *P* values were calculated via two-way Anova followed by Tukey's multiple comparisons test; **P* ≤ 0.05, ***P* ≤ 0.01, ****P* ≤ 0.001, *****P* ≤ 0.0 01; NS, not significant (*P*>0.05).

Supplementary Figure 3

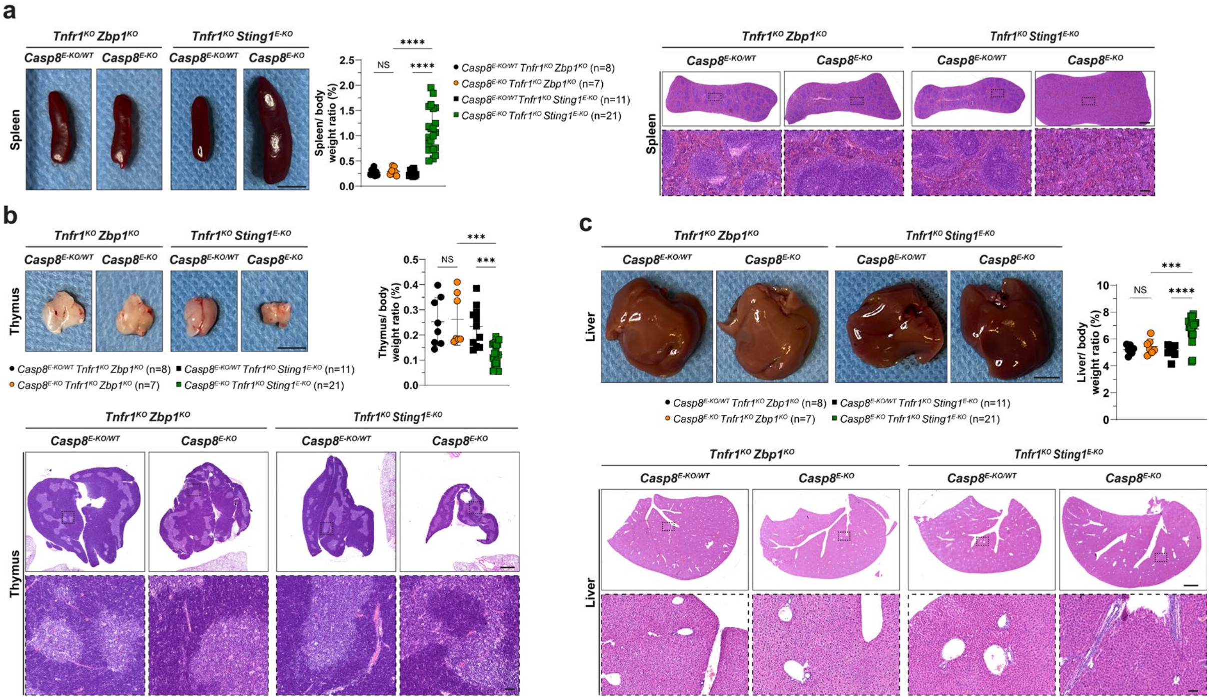

a-**c**, Representative images, graphs of organ-to-body-weight ratio and representative images of tissue sections stained with H&E of spleen (**a**), thymus (**b**) and liver (**c**) of 12-14-week-old mice with the indicated genotypes. *n* values are indicated. Scale bars: 5mm (spleen, liver and thymus images), 1 mm (bright-field images) and 100 µm (insets). Data in graphs are presented as mean ± s.e.m.; each dot represents one mouse; *P* values were calculated via two-way Anova followed by Tukey's multiple comparisons test; ****P* ≤ 0.001, *****P* ≤ 0.0001; NS, not significant (*P*>0.05).

Supplementary Figure 4

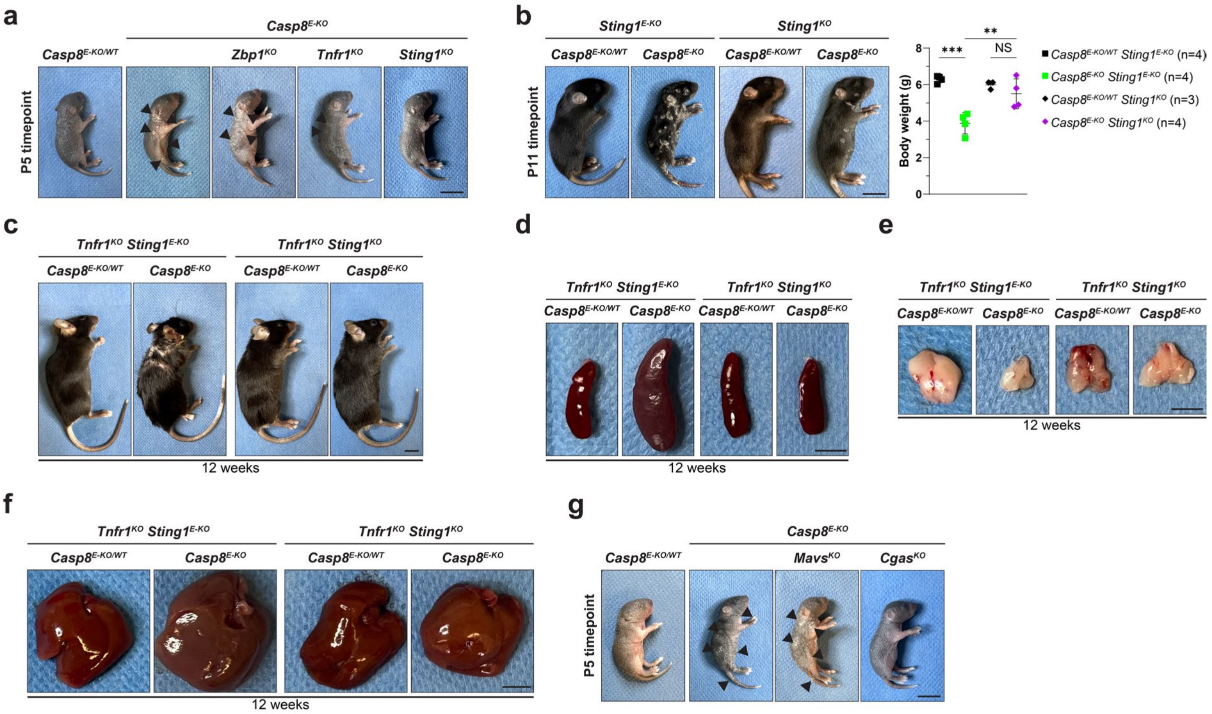

a, Representative images of mice with the indicated genotypes at P5. $n = 5$ per group. Scale bar: 1 cm. **b**, Representative images and graph of body weight of *n* mice of the indicated genotypes at the survival endpoint of $Casp8^{E\text{-}KO}$ $Sting1^{KO}$ mice (P11). Control mice included age-matched $Casp8^{E\text{-}KO/WT}$ $Sting1^{E\text{-}KO}$ and $Casp8^{E\text{-}KOWT}$ $Sting1^{KO}$ littermates. Scale bar: 1 cm. Data in graph are presented as mean ± s.e.m.; each dot represents one mouse; *P* values were calculated via two-way Anova followed by Tukey's multiple comparisons test; $^{**}P \leq 0.01$, $^{***}P \leq 0.001$; NS, not significant (P>0.05). **c**-**f**, Representative images of mice (**c**) and spleen (**d**), thymus (**e**) and liver (**f**) of 12-week-old mice of the indicated genotypes. *n*=3 per group. Control mice included age-matched $Casp8^{E\text{-}KO/WT}$ $Tnfr1^{KO}$ $Sting1^{E\text{-}KO}$ and $Casp8^{E\text{-}KO/WT}$ $Tnfr1^{KO}$ $Sting1^{KO}$ littermates. Scale bars: 1 cm (mouse images), 5mm (spleen, liver and thymus images). **g**, Representative images of mice with the indicated genotypes at P5. *n*=3 per group. Scale bar: 1 cm. In **a**, **g** arrows indicate areas with lesions.

Supplementary Figure 5

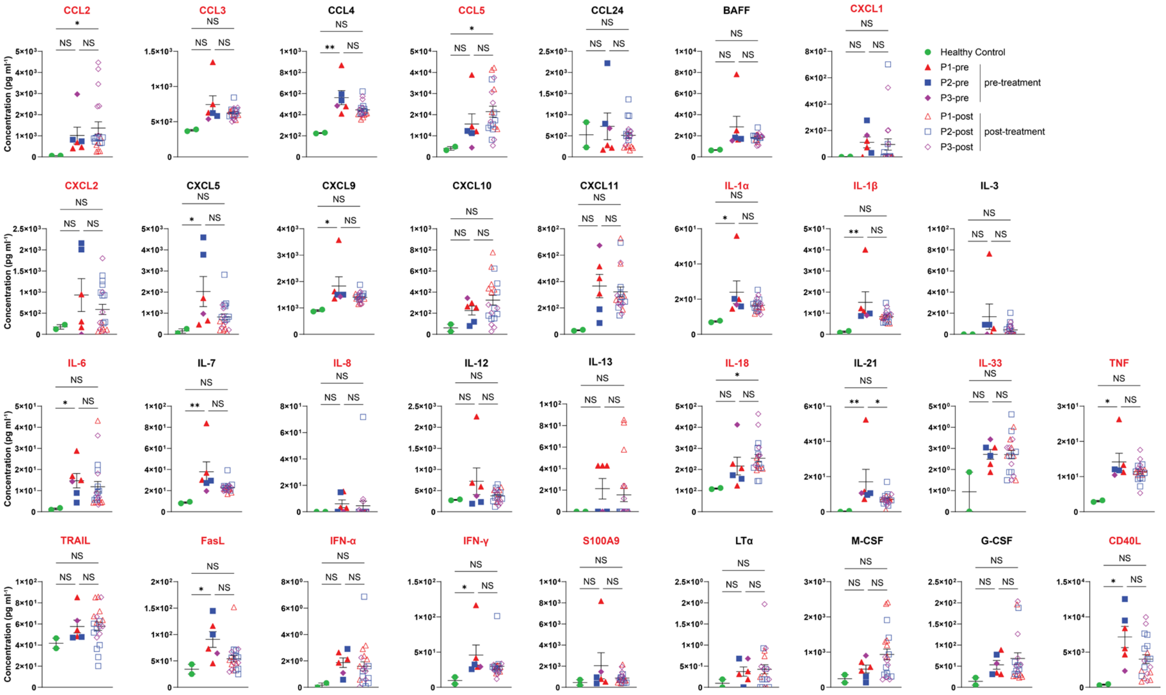

Graphs of cytokine levels (pg ml^{-1}) in plasma samples from SAVI patients, pre- and post-treatment as indicated and healthy controls, analysed via Luminex-Multiplex assay for the indicated targets. Cytokines highlighted in red denote those implicated in necroptosis, as detailed in Supplementary Table 1. Data are presented as mean ± s.e.m.; *P* values were calculated via Kruskal-Wallis test followed by Dunn's multiple comparisons test; $^{*}P \leq 0.05$, $^{**}P \leq 0.01$; NS, not significant ($P>0.05$).

Supplementary Figure 6

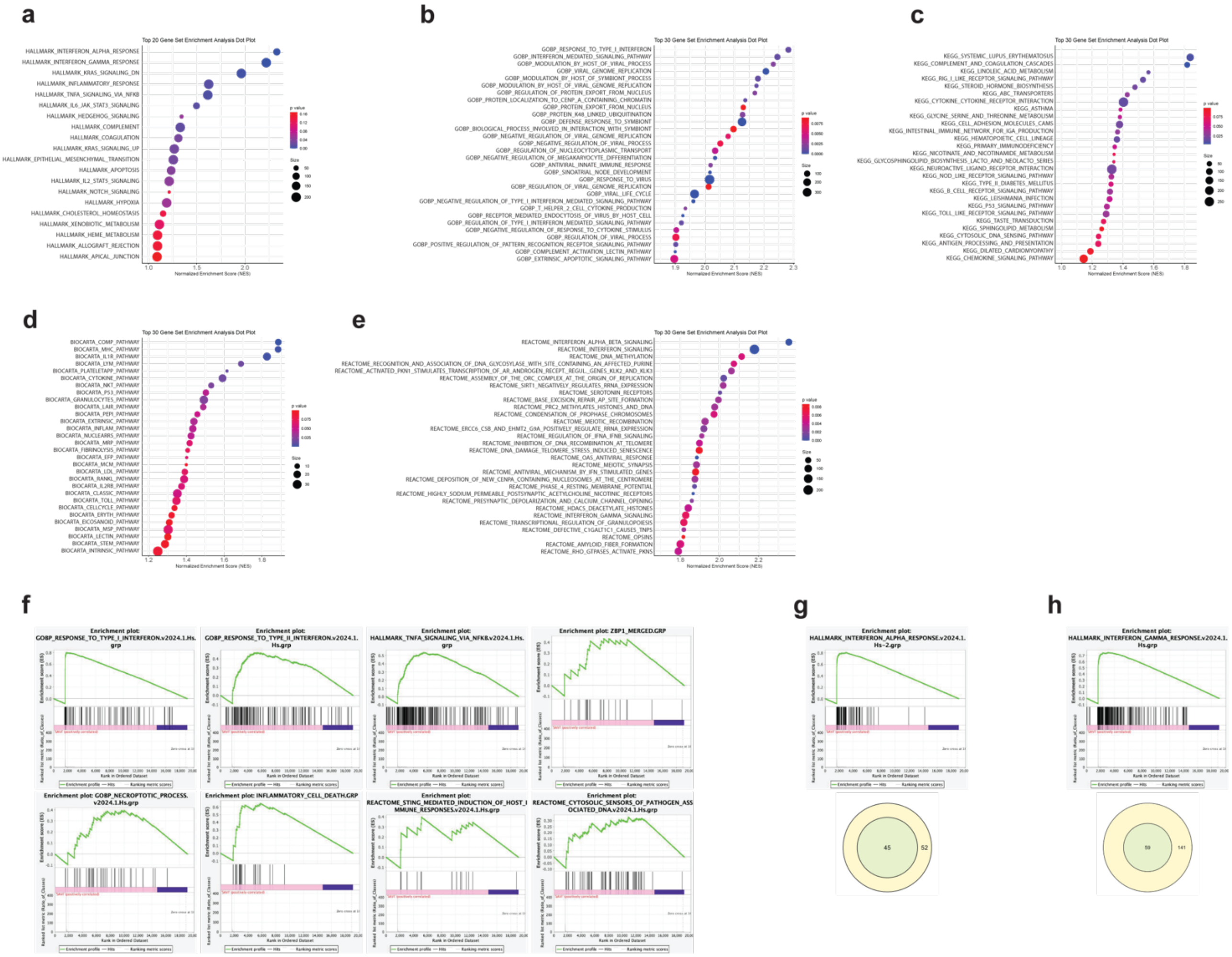

a-e, Dot plots from Gene Set Enrichment Analysis (GSEA) for top 20 or 30 upregulated pathways in SAVI IFN-high patients versus healthy donors. Hallmark (**a**), Gene Ontology Biological Process (GOBP) (**b**), KEGG (**c**), BioCarta (**d**), and Reactome (**e**) databases. Dot size represents gene count; colour intensity indicates $-\log_{10}$(*p-adj*). **f**, Enrichment plots for representative enriched pathways. Normalised Enrichment scores (NES) and positions of gene set members (black vertical lines) in the ranked gene list are shown. **g**, **h**, Enrichment plots for Type I (**g**) and Type II (**h**) interferon responses. Venn diagrams next to each plot show the overlap between upregulated interferon-related genes in IFN-high SAVI sample vs healthy control (green) and the genes in the Hallmark interferon gene sets (yellow).

Supplementary Figure 7

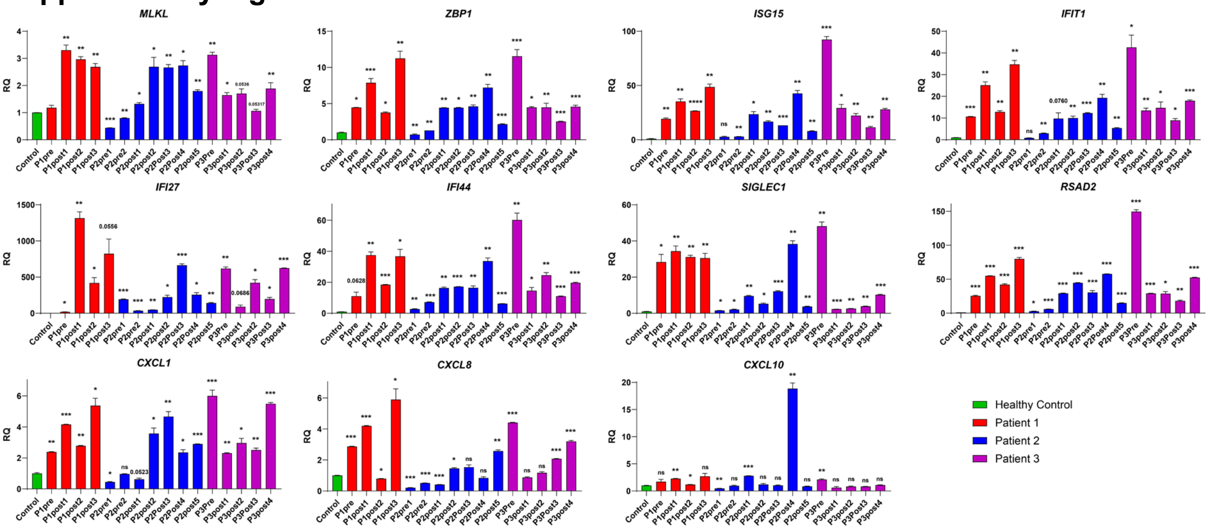

RT-qPCR analysis of mRNA expression of the indicated genes in whole blood of SAVI patients, pre- and post-treatment as indicated and healthy controls. Graphs represent relative quantification (RQ, $\log_2$) to healthy controls. Transcript levels were normalised to the housekeeper gene *HPRT.* Data are presented as mean ± s.d. from three technical replicates; *P* values were calculated via two-tailed t-test; **P* ≤ 0.05, ***P* ≤ 0.01, ****P* ≤ 0.001, *****P* ≤ 0.0001; NS, not significant (*P*>0.05).

Supplementary Figure 8

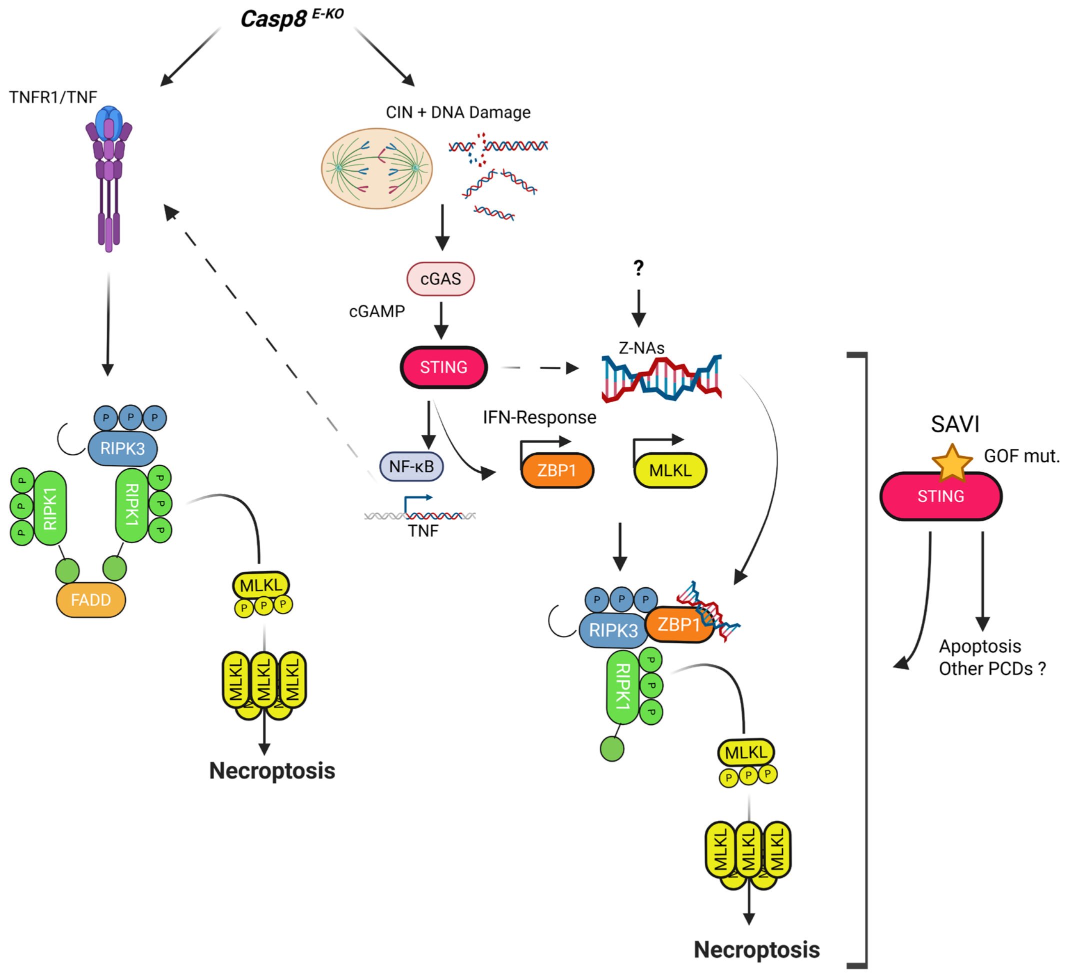

Our model illustrates the existence of two parallel, mechanistically distinct necroptotic pathways that become activated upon caspase-8 deficiency: a canonical TNF–TNFR1–FADD–RIPK1–RIPK3 axis (left), and a STING-driven, ZBP1–RIPK3–MLKL axis (right). Upon loss of Casp8, mitotic errors and DNA damage result in the accumulation of cytosolic DNA, activating the cGAS–STING pathway. STING activation induces a robust type I interferon response and NF-κB signalling, leading to transcriptional upregulation of key necroptotic effectors including ZBP1 and MLKL. Concurrently, STING promotes the accumulation or stabilization of endogenous Z-form nucleic acids (Z-NAs), which serve as ligands for ZBP1 activation. Activated ZBP1 forms a necrosome-like complex with RIPK3 and RIPK1, enabling MLKL phosphorylation and necroptotic cell death in a TNFR1-independent manner. STING also partially amplifies the canonical TNFR1 checkpoint by promoting autocrine TNF expression through NF-κB activation, thereby reinforcing TNF-mediated necroptosis. This positions STING as a central, shared regulator of both necroptotic checkpoints. Genetic evidence demonstrates that ZBP1 and STING are required for the TNFR1-independent arm of necroptosis in caspase-8-deficient keratinocytes, and that STING function extends beyond the epidermis to drive systemic inflammation via cell-extrinsic necroptosis in immune and stromal cells. Together, these data identify aberrant STING activation as a dual-function driver of necroptosis initiation and propagation, offering mechanistic insight into inflammatory disease in settings of caspase-8 loss. In the context of a STING gain-of-function (GOF) mutation, as observed in the SAVI model, necroptosis plays both pathogenic role as well as contributes to the manifestation of the disease. However, the presence of cleaved caspase-

3–positive cells in affected tissues also suggests activation of apoptotic pathways. Thus, while our findings identify necroptosis as a major contributor to SAVI pathogenesis and manifestation, they do not exclude the involvement of other programmed cell death (PCD) modalities. Created in *https://BioRender.com/l84v2zc.*

Supplementary Figure 9

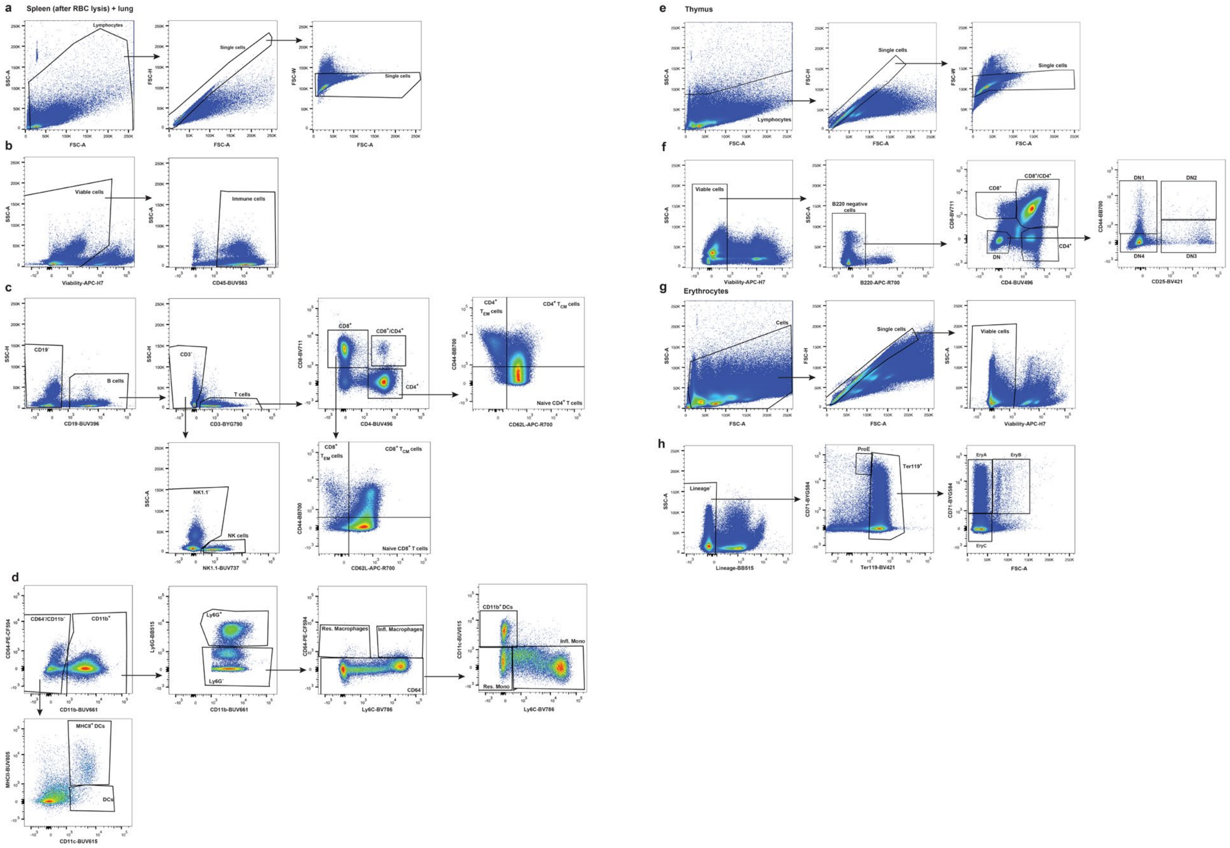

a-d, Gating strategy to determine single cell (**a)**, viable immune cell (**b**), lymphocyte (**c**) and myeloid (**d**) cell populations in the spleen and lung. **e-f**, Thymocyte population gating into single cells (**e**) and T cell developmental stages. **g-h**, Gating of erythrocyte populations from spleen homogenates.

Supplementary Table 1

	Necroptosis		Apoptosis		Pyroptosis				
Cytokine	Released	Activates	Released	Activates	Released	Activates	Immune Cell Attractant	Immune Cell Type Attracted	Pathway involvement (activated/produced by)
IL-7[54]	No	No	No	No	No	No	No	None	NF-κB, STAT5
CXCL10[55,56]	No	No	Yes	No	Yes	No	Yes	T cells, NK cells	IFNγ, NF-κB
IL-12[57]	No	No	No	No	Yes	No	Yes	NK cells, T helper cells	NF-κB, STAT4
G-CSF[58]	No	No	No	No	Yes	No	No	Neutrophils	NF-κB
LTα[56,59]	Yes	No	Yes	No	Yes	No	Yes	T cells, B cells	NF-κB, STAT3
M-CSF[56,60]	Yes	No	Yes	No	Yes	No	No	Macrophages	NF-κB
CXCL2[56,61]	Yes	No	Yes	No	Yes	No	Yes	Neutrophils, Macrophages	NF-κB, AP-1
CCL4[62]	No	No	No	No	Yes	No	Yes	T cells, NK cells	NF-κB, IRF
IL-33[63]	Indirectly	No	No	No	Yes	No	Yes	Th2 cells, ILC2	NF-κB, ST2 receptor
CXCL9[56,64]	Yes	No	No	No	Yes	No	Yes	T cells, NK cells	IFNγ, NF-κB
CCL3[56]	Yes	No	Yes	No	Yes	No	Yes	T cells, Monocytes, NK cells	NF-κB, AP-1
FasL[65]	No	Yes	No	Yes	No	No	No	None	NF-κB, FAS signaling
S100A9[56,66]	Yes	No	Yes	No	Yes	No	Yes	Neutrophils, Macrophages	NF-κB, TLR signaling
IL-18[56,67]	Yes	No	Yes	No	Yes	Yes	Yes	NK cells, T helper cells	NF-κB, Caspase-1
TRAIL[68]	No	Yes	No	Yes	No	No	No	None	NF-κB, TRAIL signaling
CCL24[69]	No	No	No	No	Yes	No	Yes	Eosinophils, Th2 cells	NF-κB, IL-4 signaling
IL-21[70]	No	No	No	No	No	No	Yes	B cells, T follicular helper cells	STAT3, NF-κB
IL-6[56,71]	Yes	No	Yes	Yes	Yes	No	Yes	T cells, B cells, Macrophages	NF-κB, STAT3
CXCL1[56,72]	Yes	No[20]	Yes	No	Yes	No	Yes	Neutrophils, Eosinophils	NF-κB, AP-1
IL-13[73]	No	No	No	No	No	No	Yes	Eosinophils, Macrophages	STAT6, NF-κB
IL-3[74]	No	No	No	No	No	No	Yes	Basophils, Eosinophils	NF-κB, STAT5
IL-1β[56,75]	Yes	No	Yes	Yes	Yes	Yes	Yes	Neutrophils, Macrophages, T cells	NF-κB, Caspase-1
IL-1α[56,76]	Yes	No	(indirectly)	No	Yes (alarmin role)	No	Yes	Neutrophils, Macrophages	NF-κB, Caspase-1
CD40L[56,77]	Indirectly	No	Indirectly	No	Indirectly	No	Yes	B cells, T cells	NF-κB, MAPK
IFN-α[78]	No	indirectly + personal comms	No	indirectly + personal comms	No	No	Yes	NK cells, Dendritic cells	STAT1, IRF
CXCL11[79]	No	No	No	No	No	No	Yes	T cells, NK cells	IFNγ, NF-κB
IL-8[56,80]	Yes	No	Yes	No	Yes	No	Yes	Neutrophils, T cells	NF-κB, AP-1
CCL2[56,81]	Yes	No	Yes	No	Yes	No	Yes	Monocytes, T cells	NF-κB, STAT3
IFN-γ[56,82]	Yes	Yes	Yes	Yes	No	No	Yes	Macrophages, T cells	STAT1, NF-κB
BAFF[83]	No	No	No	No	No	No	Yes	B cells, T cells	NF-κB
CXCL5[56,84]	Yes	No	Yes	No	Yes	No	Yes	Neutrophils, Eosinophils	NF-κB, AP-1
TNF[56,85]	Yes	Yes	No	Yes	Yes	No	Yes	Neutrophils, Macrophages, T cells	NF-κB, MAPK
CCL5[56,86]	Yes	No	Yes	No	Yes	No	Yes	T cells, NK cells, Macrophages	NF-κB, AP-1

List of cytokines and their roles in different cell death modalities.

Supplementary Table 2

IFN response	
Human Gene	Mouse Orthologue
IFITM3	*Ifitm3*
ISG15	*Isg15*
IRF7	*Irf7*
STAT1	*Stat1*
STAT2	*Stat2*
IFI35	*Ifi35*
IFI27	*Ifi27*
IFI44	*Ifi44*
GBP3	*Gbp3*
GBP5	*Gbp5*
SIGLEC1	*siglec1*
MX1	*Mx1*
MX2	*Mx2*
USP18	*Usp18*
RSAD2	*Rsad2*
RTP4	*Rtp4*
BST2	*Bst2*
XAF1	*Xaf1*
PARP9	*Parp9*
PARP12	*Parp12*
PARP14	*Parp14*
HERC5	*Herc6*
TAP1	*Tap1*
TRIM5	*Trim5*
EPSTI1	*Epsti1*
SAMD9L	*Samd9l*
TRIM25	*trim25*

Necroptosis	
Human Gene	Mouse Orthologue
MLKL	*Mlkl*
ZBP1	*Zbp1*
EIF2AK2	*Eif2ak2*
JUNB	*Junb*

Nucleic acid sensing	
Human Gene	Mouse Orthologue
DDX58	*Ddx58*
TREX1	*Trex1*
IFIH1	*Ifih1*
IFIT1	*Ifit1*
IFIT3	*Ifit3*
ZC3HAV1	*Zc3hav1*
DDX60	*Ddx60*
OASL	*Oasl1*
OAS1	*Oas1a*
	Oas1b
	Oas1c
	Oas1g
OAS2	*Oas2*
OAS3	*Oas3*
HELZ2	*Helz2*
DHX58	*Dhx58*

Shared upregulated genes in IFN-high SAVI and *Casp8*$^{E\text{-}KO}$ mice, categorized by IFN response, nucleic acid sensing, and necroptosis. Table presents selected differentially upregulated genes categorised into three groups: IFN response, nucleic acid sensing, and necroptosis. These genes were identified as upregulated in IFN-high SAVI samples relative to controls and show overlap with genes upregulated in *Casp8*$^{E\text{-}KO}$ mice compared to their respective controls.

Supplementary Table 3

Antibody	Identifier	Source	Dilution
FADD	05-486	Millipore	1:1000
RIPK1	3493	Cell Signalling Technology	1:1000
	610459	BD Biosciences	1:500
pRIPK1 (S166)	31122	Cell Signalling Technology	1:500
RIPK3	95702	Cell Signalling Technology	1:1000
pRIPK3 (T231/S232)	91702	Cell Signalling Technology	1:500
MLKL	MABC604	Millipore	1:1000
pMLKL (S345)	37333	Cell Signalling Technology	1:1000
ZBP1	AG-20B-0010-C100	Adipogen	1:1000
pSTAT1 (Y701)	5483	Cell Signalling Technology	1:250
TBK1	3013	Cell Signalling Technology	1:1000
pTBK1 (S172)	72971	Cell Signalling Technology	1:500
caspase-8	ALX-804-447	Enzo Life Sciences	1:1000
STING	13647	Cell Signalling Technology	1:1000
Total OXPHOS	ab110413	Abcam	1:500
GAPDH	G9545	Sigma-Aldrich	1:10000

anti-mouse IgG	JIM-115-035-174	Jackson ImmunoResearch	1:5000
anti-rat IgG	JIM-112-035-175	Jackson ImmunoResearch	1:5000
anti-rabbit IgG	JIM-211-032-171	Jackson ImmunoResearch	1:5000

List of antibodies used for Western Blot analysis.

Supplementary Table 4

Antibody	Identifier	Source	Dilution
pMLKL (S345)	37333	Cell Signalling Technology	1:2000
Cl. CASP3 (skin)	9664	Cell Signalling Technology	1:100
Cl. CASP3 (other tissues)	9661	Cell Signalling Technology	1:200
ZBP1	AG-20B-0010-C100	Adipogen	1:800
STING	13647	Cell Signalling Technology	1:200
pSTAT1 (Y701)	9167	Cell Signalling Technology	1:50
CD3	ab5690	Abcam	1:1400
Ly6G	87048	Cell Signalling Technology	1:100
pHH3 (S10)	06-570	Sigma-Aldrich	1:500
γH2AX (S139)	9718	Cell Signalling Technology	1:50
F4/80	MCA497R	BioRad	1:50
CD45	550539	BD Biosciences	1:200
Keratin-6A	905701	BioIrgend	1:400
Keratin-10	ab9026	Abcam	1:200
Keratin-14	905304	Biolegend	1:400
goat anti-rabbit	MP-7451	Vector Laboratories	kit
goat anti-rat	MP-7444-15	Vector Laboratories	kit
mouse-on-mouse	MP-2400	Vector Laboratories	kit
biotinylated goat anti-rabbit	BA-1000	Vector Laboratories	1:200
biotinylated goat anti-rat	BA-9400	Vector Laboratories	1:200
goat anti-rabbit Alexa Fluor Plus 488	A32731	Thermo Fisher Scientific	1:400
goat anti-mouse Alexa Fluor Plus 594	A32742	Thermo Fisher Scientific	1:400

Antibodies used for *in situ* immunohistochemistry and immunofluorescence.

Supplementary Table 5

Target gene	Forward primer	Reverse primer
m*Isg15*	GTGCTCCAGGACGGTCTTAC	CTCGCTGCAGTTCTGTACCA
m*Tnf*	CAGGCGGTGCCTATGTCTC	CGATCACCCCGAAGTTCAGTAG
m*S100a9*	TGGTGGAAGCACAGTTGGCAAC	CAGCATCATACACTCCTCAAAGC
m*Mlkl*	AATTGTACTCTGGGAAATTGCCA	TCTCCAAGATTCCGTCCACAG
m*Ripk3*	TCTGTCAAGTTATGGCCTACTGG	GGAACACGACTCCGAACCC
m*Ripk1*	GACTGTGTACCCTTACCTCCGA	CACTGCGATCATTCTCGTCCTG
m*Ifnβ*	CAGCTCCAAGAAAGGACGAAC	GGCAGTGTAACTCTTCTGCAT
m*Ifnγ*	CGGCACAGTCATTGAAAGCCTA	GTTGCTGATGGCCTGATTGTC
m*Gapdh*	CTCCCACTCTTCCACCTTCG	GCCTCTCTTGCTCAGTGTCC

h*MLKL*	TAATTCTGAGAAGATCCGCAAG	GGAGAGTTTCTTTAAGATTTCAT
h*ZBP1*	GCAAACTCCGAAGCCATCCAGA	CCAAGTTGAGGAATCACCTGGTG
h*ISG15*	GCGAACTCATCTTTGCCAGTA	CCAGCATCTTCACCGTCAG
h*IFIT1*	CTGGCAGAAGCCCAGACTTACC	AGGCCCATCCTTCCTCACAGTCT
h*IFI27*	CGTCCTCCATAGCAGCCAAGAT	ACCCAATGGAGCCCAGGATGAA
h*IFI44*	TGGTACATGTGGCTTTGCTC	CCACCGAGATGTCAGAAAGAG
h*SIGLEC1*	ACCTGGAGGAAACTGACAGTGG	CTCAGTGTCACTGCCTGTCCTT
h*RSAD2*	CCAGTGCAACTACAAATGCGGC	CGGTCTTGAAGAAATGGCTCTCC
h*CXCL1*	TCCTGCATCCCCCATAGTTA	CTTCAGGAACAGCCACCAGT
h*CXCL8*	TCTGGCAACCCTAGTCTGCT	AAACCAAGGCACAGTGGAAC
h*CXCL10*	GTGGATGTTCTGACCCTGCT	GAGGATGGCAGTGGAAGTCC
h*HPRT*	AGCCAGACTTTGTTGGATTTG	TTTACTGGCGATGTCAATAGG

Primer sequences used for RT-qPCR in tissue samples.

Supplementary Table 6

Antibody	Fluorochrome	Identifier	Source	Dilution
B220	FITC	103205	BioLegend	1:200
B220	AF700	103232	BioLegend	1:200
CD11b	BUV661	612977	BD Biosciences	1:250
CD11b	FITC	101205	BioLegend	1:200
CD11c	BV605	117334	BioLegend	1:200
CD19	BUV395	565965	BD Biosciences	1:100
CD25	BV421	562606	BD Biosciences	1:100
CD3	PE-Cy7	100219	BioLegend	1:200
CD3	FITC	100203	BioLegend	1:200
CD4	BUV496	612952	BD Biosciences	1:200
CD44	BB700	566507	BD Biosciences	1:200
CD45	BUV563	612924	BD Biosciences	1:250
CD5	FITC	553020	BD Biosciences	1:100
CD62L	AF700	104441	BioLegend	1:100
CD64	PE-Dazzle	139319	BioLegend	1:100
CD69	APC	560689	BD Biosciences	1:100
CD71	PE	113807	BioLegend	1:100
CD8	BV711	563046	BD Biosciences	1:100
GR-1	FITC	108405	BioLegend	1:200
Ly6C	BV785	128041	BioLegend	1:150
Ly6G	FITC	551460	BD Biosciences	1:100
MHCII	BUV805	748844	BD Biosciences	1:100
NK1.1	BUV737	741715	BD Biosciences	1:100
TCRβ	PE	553172	BD Biosciences	1:100
Ter119	BV421	116233	BioLegend	1:200

Antibodies for flow cytometric analysis.

Supplementary Table 7

Target gene	Forward primer	Reverse primer
mt*Dloop*	AATCTACCATCCTCCGTGAAACC	TCAGTTTAGCTACCCCCAAGTTTAA
mt*Co1*	GCAGGAGCATCAGTAGACCTAAC	GGAGTTTGATACTGTGTTATGGCTGG
mt*Co3*	GACGTAATTCGTGAAGGAACCTACC	GATAGAACGCTCAGAAGAATCCTGC
mt*Nd2*	GCATGAGGAGGACTTAACCAAACAC	GAGGTTGAGTAGAGTGAGGGATGG
mt*Nd5*	GGCCTATTAATCGCAGCTACAGG	GTAGTAGTGCTGAAACTGGTGTAGG
mt*Atp6*	CCTTCCACAAGGAACTCCAATTTCAC	CTAGAGTAGCTCCTCCGATTAGGTG
mt*Rnr2*	GGGATAACAGCGCAATCCTA	GATTGCTCCGGTCTGAACTC
Actb	CATTGCTGACAGGATGCAGAAGG	TGCTGGAAGGTGGACAGTGAGG

Primer sequences targeting mtDNA-encoded genes used for RT–qPCR.

Supplementary Table 8

Target gene	Forward primer	Reverse primer
m*Zbp1*	TGTTGACTTGAGCACAGGAG	TTCAGGCGGTAAAGGACTTG
m*Isg15*	CTAGAGCTAGAGCCTGCAG	AGTTAGTCACGGACACCAG
m*Usp18*	GAGAGGACCATGAAGAGGA	TAAACCAACCAGACCATGAG
m*Rsad2*	CCCGTGAGTGTCAACTACCAC	GCCCAAGTATTCACCCCTGTC
m*Ifit1*	CAAGGCAGGTTTCTGAGGAG	GACCTGGTCACCATCAGCAT
m*Hprt*	TCCTCCTCAGACCGCTTTT	CATAACCTGGTTCATCATCGC

Primer sequences used for RT-qPCR in MEFs.

Supplementary Table 9

Vials code	Patient ID	Mutation Status	Processing date	Sample collected	PRE/POST TREATMENT	Disease Response (CR/PR/SD)	Assessment	Notes
I-3200P	1	c.[463G>A]; p.[Val155Met]	29/11/2017	Plasma	PRE-treatment	Active disease		First visit
I-3239P	1		13/12/2017	Plasma	PRE-treatment	SD		
I-3445P	1		22/02/2018	Plasma	PRE-treatment	SD		
I-3851P	1		06/07/2018	Plasma	POST-treatment	PR		
I-4577P	1		04/02/2019	Plasma	POST-treatment	PR		
I-5022P	1		04/06/2019	Plasma	POST-treatment	PR		
I-5945P	1		10/01/2020	Plasma	POST-treatment	PR		
I-7874P	1		14/04/2021	Plasma	POST-treatment	PR		
I-9675P	1		21/03/2022	Plasma	POST-treatment	PR		
I-3200TT	1		29/11/2017	RNA	PRE-treatment	Active disease		First visit
I-3445TT	1		22/02/2018	RNA	PRE-treatment	SD		
I-3851TT	1		06/07/2018	RNA	POST-treatment	PR		
I-4577TT	1		04/02/2019	RNA	POST-treatment	PR		
I-5945TT	1		10/01/2020	RNA	POST-treatment	PR		
I-2202P	2	c.[461A>G]; p.[Asn154Ser]	27/10/2016	Plasma	PRE-treatment	Active disease		
I-2406P	2		26/01/2017	Plasma	PRE-treatment	SD		
I-3047P	2		04/10/2017	Plasma	POST-treatment	PR		
I-3214P	2		05/12/2017	Plasma	POST-treatment	PR		
I-4001P	2		30/08/2018	Plasma	POST-treatment	PR		
I-4490P	2		15/01/2019	Plasma	POST-treatment	PR		
I-4631P	2		12/02/2019	Plasma	POST-treatment	PR		
I-5934P	2		09/01/2020	Plasma	POST-treatment	PR		
I-7616P	2		15/02/2021	Plasma	POST-treatment	PR		
I-9677P	2		21/03/2022	Plasma	POST-treatment	PR		
I-2193Pax	2		26/10/2016	RNA	PRE-treatment	Active disease		
I-2406Pax	2		26/01/2017	RNA	PRE-treatment	SD		
I-2491Pax	2		01/03/2017	RNA	POST-treatment	PR		
I-3047Pax	2		04/10/2017	RNA	POST-treatment	PR		
I-3214Pax	2		05/12/2017	RNA	POST-treatment	PR		
I-3214TT	2		05/12/2017	RNA	POST-treatment	PR		
I-4001TT	2		30/08/2018	RNA	POST-treatment	PR		
I-7953P	3	c.[463G>A]; p.[Val155Met]	27/04/2021	Plasma	PRE-treatment	Active disease		
I-8045P	3		12/05/2021	Plasma	POST-treatment	SD		
I-8304P	3		06/07/2021	Plasma	POST-treatment	PR		
I-8913P	3		03/11/2021	Plasma	POST-treatment	PR		
I-9288P	3		27/01/2022	Plasma	POST-treatment	PR		
I-9810P	3		12/04/2022	Plasma	POST-treatment	PR		
I-10153P	3		13/06/2022	Plasma	POST-treatment	PR		
I-7953TT	3		27/04/2021	RNA	PRE-treatment	Active disease		
I-8045TT	3		12/05/2021	RNA	POST-treatment	SD		
I-8304TT	3		06/07/2021	RNA	POST-treatment	PR		
I-9288TT	3		27/01/2022	RNA	POST-treatment	PR		
I-10153TT	3		13/06/2022	RNA	POST-treatment	PR		

Clinical characterization of patients and patient sample information.

54 Minton, K. Inflammasomes: ubiquitin lines up for inflammasome activity. *Nat Rev Immunol* **14**, 580-581, doi:10.1038/nri3730 (2014).
55 Chen, L. *et al.* Genetic Drivers of Epigenetic and Transcriptional Variation in Human Immune Cells. *Cell* **167**, 1398-1414 e1324, doi:10.1016/j.cell.2016.10.026 (2016).
56 Tanzer, M. C. *et al.* Quantitative and Dynamic Catalogs of Proteins Released during Apoptotic and Necroptotic Cell Death. *Cell Rep* **30**, 1260-1270 e1265, doi:10.1016/j.celrep.2019.12.079 (2020).
57 Smith, N. L. *et al.* Developmental Origin Governs CD8(+) T Cell Fate Decisions during Infection. *Cell* **174**, 117-130 e114, doi:10.1016/j.cell.2018.05.029 (2018).
58 Corey, S. J. *et al.* Granulocyte colony-stimulating factor receptor signaling involves the formation of a three-component complex with Lyn and Syk protein-tyrosine kinases. *Proc Natl Acad Sci U S A* **91**, 4683-4687, doi:10.1073/pnas.91.11.4683 (1994).
59 Etemadi, N. *et al.* Lymphotoxin alpha induces apoptosis, necroptosis and inflammatory signals with the same potency as tumour necrosis factor. *FEBS J* **280**, 5283-5297, doi:10.1111/febs.12419 (2013).
60 Yi, L. *et al.* Macrophage colony-stimulating factor and its role in the tumor microenvironment: novel therapeutic avenues and mechanistic insights. *Front Oncol* **14**, 1358750, doi:10.3389/fonc.2024.1358750 (2024).
61 Sinha, S. K. *et al.* Local M-CSF (Macrophage Colony-Stimulating Factor) Expression Regulates Macrophage Proliferation and Apoptosis in Atherosclerosis. *Arterioscler Thromb Vasc Biol* **41**, 220-233, doi:10.1161/ATVBAHA.120.315255 (2021).
62 Bystry, R. S., Aluvihare, V., Welch, K. A., Kallikourdis, M. & Betz, A. G. B cells and professional APCs recruit regulatory T cells via CCL4. *Nat Immunol* **2**, 1126-1132, doi:10.1038/ni735 (2001).
63 Shlomovitz, I. *et al.* Necroptosis directly induces the release of full-length biologically active IL-33 in vitro and in an inflammatory disease model. *FEBS J* **286**, 507-522, doi:10.1111/febs.14738 (2019).
64 Veerman, K., Tardiveau, C., Martins, F., Coudert, J. & Girard, J. P. Single-Cell Analysis Reveals Heterogeneity of High Endothelial Venules and Different Regulation of Genes Controlling Lymphocyte Entry to Lymph Nodes. *Cell Rep* **26**, 3116-3131 e3115, doi:10.1016/j.celrep.2019.02.042 (2019).
65 Huang, D. C. *et al.* Activation of Fas by FasL induces apoptosis by a mechanism that cannot be blocked by Bcl-2 or Bcl-x(L). *Proc Natl Acad Sci U S A* **96**, 14871-14876, doi:10.1073/pnas.96.26.14871 (1999).
66 Christmann, C. *et al.* Interleukin 17 Promotes Expression of Alarmins S100A8 and S100A9 During the Inflammatory Response of Keratinocytes. *Front Immunol* **11**, 599947, doi:10.3389/fimmu.2020.599947 (2020).
67 Chan, A. H. & Schroder, K. Inflammasome signaling and regulation of interleukin-1 family cytokines. *J Exp Med* **217**, doi:10.1084/jem.20190314 (2020).
68 Montinaro, A. & Walczak, H. Harnessing TRAIL-induced cell death for cancer therapy: a long walk with thrilling discoveries. *Cell Death Differ* **30**, 237-249, doi:10.1038/s41418-022-01059-z (2023).
69 Diny, N. L. *et al.* Macrophages and cardiac fibroblasts are the main producers of eotaxins and regulate eosinophil trafficking to the heart. *Eur J Immunol* **46**, 2749-2760, doi:10.1002/eji.201646557 (2016).
70 Spolski, R. & Leonard, W. J. Interleukin-21: a double-edged sword with therapeutic potential. *Nat Rev Drug Discov* **13**, 379-395, doi:10.1038/nrd4296 (2014).

71 Shkarina, K. *et al.* Optogenetic activators of apoptosis, necroptosis, and pyroptosis. *J Cell Biol* **221**, doi:10.1083/jcb.202109038 (2022).

72 Han, K. Q. *et al.* Targeted silencing of CXCL1 by siRNA inhibits tumor growth and apoptosis in hepatocellular carcinoma. *Int J Oncol* **47**, 2131-2140, doi:10.3892/ijo.2015.3203 (2015).

73 Terabe, M., Park, J. M. & Berzofsky, J. A. Role of IL-13 in regulation of anti-tumor immunity and tumor growth. *Cancer Immunol Immunother* **53**, 79-85, doi:10.1007/s00262-003-0445-0 (2004).

74 Broughton, S. E. *et al.* The GM-CSF/IL-3/IL-5 cytokine receptor family: from ligand recognition to initiation of signaling. *Immunol Rev* **250**, 277-302, doi:10.1111/j.1600-065X.2012.01164.x (2012).

75 Wertman, R. S. *et al.* Distinct sequential death complexes regulate pyroptosis and IL-1beta release in response to Yersinia blockade of immune signaling. *Sci Adv* **10**, eadl3629, doi:10.1126/sciadv.adl3629 (2024).

76 Cohen, I. *et al.* Differential release of chromatin-bound IL-1alpha discriminates between necrotic and apoptotic cell death by the ability to induce sterile inflammation. *Proc Natl Acad Sci U S A* **107**, 2574-2579, doi:10.1073/pnas.0915018107 (2010).

77 Qiu, X., Klausen, C., Cheng, J. C. & Leung, P. C. CD40 ligand induces RIP1-dependent, necroptosis-like cell death in low-grade serous but not serous borderline ovarian tumor cells. *Cell Death Dis* **6**, e1864, doi:10.1038/cddis.2015.229 (2015).

78 Takaoka, A. *et al.* Integration of interferon-alpha/beta signalling to p53 responses in tumour suppression and antiviral defence. *Nature* **424**, 516-523, doi:10.1038/nature01850 (2003).

79 Petkovic, V., Moghini, C., Paoletti, S., Uguccioni, M. & Gerber, B. I-TAC/CXCL11 is a natural antagonist for CCR5. *J Leukoc Biol* **76**, 701-708, doi:10.1189/jlb.1103570 (2004).

80 Harada, A. *et al.* Essential involvement of interleukin-8 (IL-8) in acute inflammation. *J Leukoc Biol* **56**, 559-564 (1994).

81 Deshmane, S. L., Kremlev, S., Amini, S. & Sawaya, B. E. Monocyte chemoattractant protein-1 (MCP-1): an overview. *J Interferon Cytokine Res* **29**, 313-326, doi:10.1089/jir.2008.0027 (2009).

82 Bertheloot, D., Latz, E. & Franklin, B. S. Necroptosis, pyroptosis and apoptosis: an intricate game of cell death. *Cell Mol Immunol* **18**, 1106-1121, doi:10.1038/s41423-020-00630-3 (2021).

83 Mackay, F., Schneider, P., Rennert, P. & Browning, J. BAFF AND APRIL: a tutorial on B cell survival. *Annu Rev Immunol* **21**, 231-264, doi:10.1146/annurev.immunol.21.120601.141152 (2003).

84 Sokulsky, L. A. *et al.* A Critical Role for the CXCL3/CXCL5/CXCR2 Neutrophilic Chemotactic Axis in the Regulation of Type 2 Responses in a Model of Rhinoviral-Induced Asthma Exacerbation. *J Immunol* **205**, 2468-2478, doi:10.4049/jimmunol.1901350 (2020).

85 Brenner, D., Blaser, H. & Mak, T. W. Regulation of tumour necrosis factor signalling: live or let die. *Nat Rev Immunol* **15**, 362-374, doi:10.1038/nri3834 (2015).

86 Appay, V. & Rowland-Jones, S. L. RANTES: a versatile and controversial chemokine. *Trends Immunol* **22**, 83-87, doi:10.1016/s1471-4906(00)01812-3 (2001).
