# Supplementary material for: STING induces ZBP1-mediated necroptosis independently of TNFR1 and FADD
Source: Nature. 2025 Aug 20;647(8090):735–46. doi: 10.1038/s41586-025-09536-4 (PMC12629989; doi:10.1038/s41586-025-09536-4)
Supplement: Supplementary file 2 — Reporting Summary [file 41586_2025_9536_MOESM2_ESM.pdf]

nature portfolio

Corresponding author(s): Liccardi Gianmaria

Last updated by author(s): 27/07/2025

# Reporting Summary

Nature Portfolio wishes to improve the reproducibility of the work that we publish. This form provides structure for consistency and transparency in reporting. For further information on Nature Portfolio policies, see our Editorial Policies and the Editorial Policy Checklist.

## Statistics

For all statistical analyses, confirm that the following items are present in the figure legend, table legend, main text, or Methods section.

| n/a | Confirmed | |
|---|---|---|
| ☐ | ☒ | The exact sample size ($n$) for each experimental group/condition, given as a discrete number and unit of measurement |
| ☐ | ☒ | A statement on whether measurements were taken from distinct samples or whether the same sample was measured repeatedly |
| ☐ | ☒ | The statistical test(s) used AND whether they are one- or two-sided<br>*Only common tests should be described solely by name; describe more complex techniques in the Methods section.* |
| ☐ | ☒ | A description of all covariates tested |
| ☐ | ☒ | A description of any assumptions or corrections, such as tests of normality and adjustment for multiple comparisons |
| ☐ | ☒ | A full description of the statistical parameters including central tendency (e.g. means) or other basic estimates (e.g. regression coefficient) AND variation (e.g. standard deviation) or associated estimates of uncertainty (e.g. confidence intervals) |
| ☐ | ☒ | For null hypothesis testing, the test statistic (e.g. $F$, $t$, $r$) with confidence intervals, effect sizes, degrees of freedom and $P$ value noted<br>*Give P values as exact values whenever suitable.* |
| ☒ | ☐ | For Bayesian analysis, information on the choice of priors and Markov chain Monte Carlo settings |
| ☒ | ☐ | For hierarchical and complex designs, identification of the appropriate level for tests and full reporting of outcomes |
| ☒ | ☐ | Estimates of effect sizes (e.g. Cohen's $d$, Pearson's $r$), indicating how they were calculated |

*Our web collection on statistics for biologists contains articles on many of the points above.*

## Software and code

Policy information about availability of computer code

| | |
|---|---|
| Data collection | n/a |
| Data analysis | n/a |

For manuscripts utilizing custom algorithms or software that are central to the research but not yet described in published literature, software must be made available to editors and reviewers. We strongly encourage code deposition in a community repository (e.g. GitHub). See the Nature Portfolio guidelines for submitting code & software for further information.

## Data

Policy information about availability of data

All manuscripts must include a data availability statement. This statement should provide the following information, where applicable:

- Accession codes, unique identifiers, or web links for publicly available datasets
- A description of any restrictions on data availability
- For clinical datasets or third party data, please ensure that the statement adheres to our policy

Sequencing Data from patient samples are available upon request. Sequencing data relative to the mouse model as indicated in the manuscript are deposited. Accession number is available in the Data availability paragraph contained in the Material and Methods section.

## Research involving human participants, their data, or biological material

Policy information about studies with human participants or human data. See also policy information about sex, gender (identity/presentation), and sexual orientation and race, ethnicity and racism.

| | |
|---|---|
| Reporting on sex and gender | 3 female patiens |
| Reporting on race, ethnicity, or other socially relevant groupings | Caucasian White |
| Population characteristics | N/A no clinical trial performed |
| Recruitment | Patients were recruited on the basis of their positive screening for STING activating mutations |
| Ethics oversight | The study was approved by the Ethics Committee of Bambino Gesù Children's Hospital (protocol number 3086 ) and was conducted in accordance with the Declaration of Helsinki. Written informed consent was obtained from all participants and/or their parents or legal guardians. |

Note that full information on the approval of the study protocol must also be provided in the manuscript.

# Field-specific reporting

Please select the one below that is the best fit for your research. If you are not sure, read the appropriate sections before making your selection.

☒ Life sciences ☐ Behavioural & social sciences ☐ Ecological, evolutionary & environmental sciences

For a reference copy of the document with all sections, see nature.com/documents/nr-reporting-summary-flat.pdf

# Life sciences study design

All studies must disclose on these points even when the disclosure is negative.

| | |
|---|---|
| Sample size | All experiments were repeated at least three times with at least 2 biological replicates for in vitro experiments, while in vivo experiments included at least 3 biological replicates. All mouse survival cohorts are based on a minimum of at least 5 mice per genotype. n values represent number of mice and are accurately reported throughout all figure panels. |
| Data exclusions | no data was excluded |
| Replication | everything was replicated at least three times |
| Randomization | No randomisation needed |
| Blinding | stainings were performed blindly when possible |

# Reporting for specific materials, systems and methods

We require information from authors about some types of materials, experimental systems and methods used in many studies. Here, indicate whether each material, system or method listed is relevant to your study. If you are not sure if a list item applies to your research, read the appropriate section before selecting a response.

## Materials & experimental systems

| n/a | Involved in the study | |
|---|---|---|
| ☐ | ☒ | Antibodies |
| ☐ | ☒ | Eukaryotic cell lines |
| ☒ | ☐ | Palaeontology and archaeology |
| ☐ | ☒ | Animals and other organisms |
| ☒ | ☐ | Clinical data |
| ☒ | ☐ | Dual use research of concern |
| ☒ | ☐ | Plants |

## Methods

| n/a | Involved in the study | |
|---|---|---|
| ☒ | ☐ | ChIP-seq |
| ☐ | ☒ | Flow cytometry |
| ☒ | ☐ | MRI-based neuroimaging |

## Antibodies

| | |
|---|---|
| Antibodies used | Primary antibodies used: FADD (05-486, Millipore, 1:1000), RIPK1 (3493, Cell Signalling Technology, 1:1000 and 610459, BD |

Antibodies used

Biosciences, 1:500), pRIPK1-S166 (31122, Cell Signalling Technology, 1:500), RIPK3 (95702, Cell Signalling Technology, 1:1000), pRIPK3 (91702, Cell Signalling Technology, 1:500), MLKL (MABC604, Millipore, 1:1000), pMLKL-S345 (37333, Cell Signalling Technology, 1:1000), ZBP1 (AG-20B-0010-C100, Adipogen, 1:1000), pSTAT1-Y701 (5483, Cell Signalling Technology, 1:500), TBK1 (3013, Cell Signalling Technology, 1:1000), pTBK1-S172 (72971, Cell Signalling Technology, 1:500), Caspase-8 (ALX-804-447, Enzo Life Sciences, 1:1000) GAPDH (G9545, Sigma-Aldrich, 1:10000), Total OXPHOS (ab110413, Abcam, 1:500), CD3 (ab5690, Abcam, 1:1400), Ly6G (87048, Cell Signalling Technology, 1:100), p-HH3 (06-570, Sigma-Aldrich, 1:500), γ-H2AX (9718, Cell Signalling Technology,1:50), c-Casp3 (9664 for skin, 9661 for the rest of the tissues, Cell Signalling Technology, 1:100), F4/80 (MCA497R, BioRad, 1:50), CD45 (550539, BD Biosciences, 1:200), Keratin-6A (905701, BioLegend, 1:400), Keratin-10 (ab9026, Abcam, 1:200) and Keratin-14 (905304, BioLegend, 1:400), Z-DNA/Z-RNA [Z22] (Ab00783-23.0, Absolute Antibody), TFAM (ABE483, EMD Millipore; 1:200) and DNA (CBL186, EMD Millipore; 1:100), B220-FITC (103205, BioLegend, 1:200), B220-AF700 (103232, BioLegend, 1:200), CD11b-BUV661 (612977, BD Biosciences, 1:250), CD11b-FITC (101205, BioLegend, 1:200), CD11c-BV605 (117334, BioLegend, 1:200), CD19-BUV395 (565965, BD Biosciences, 1:100), CD25-BV421 (562606, BD Biosciences, 1:100), CD3-PE-Cy7 (100219, BioLegend, 1:200), CD3-FITC (100203, BioLegend, 1:200), CD4-BUV496 (612952, BD Biosciences, 1:200), CD44-BB700 (566507, BD Biosciences, 1:200), CD45-BUV563 (612924, BD Biosciences, 1:250), CD5-FITC (553020, BD Biosciences, 1:100), CD62L-AF700 (104441, BioLegend, 1:100), CD64-PE-Dazzle (139319, BioLegend, 1:100), CD69-APC (560689, BD Biosciences, 1:100), CD71-PE (113807, BioLegend, 1:100), CD8-BV711 (563046, BD Biosciences, 1:100), GR-1-FITC (108405, BioLegend, 1:200), Ly6C-BV785 (128041, BioLegend, 1:150), Ly6G-FITC (551460, BD Biosciences, 1:100), MHCII-BUV805 (748844, BD Biosciences, 1:100), NK1.1-BUV737 (741715, BD Biosciences, 1:100), TCRβ-PE (553172, BD Biosciences, 1:100) and Ter119-BV421 (116233, BioLegend, 1:200).

Validation

All antibodies used have all been published before  in our field or tested in presence of knock out cells or functionally validated withing the context of this paper. Antibodies recently published to be used for any staining have been also referenced throughout the manuscript.

## Eukaryotic cell lines

Policy information about cell lines and Sex and Gender in Research

Cell line source(s)

ATCC for human cell lines, for mouse all lines were derived as primary MEFs from E13.5 embryos obtained from time mating of mice bred in the study , and genotyped  for desired genotype or previously described. The cell lines were subsequently immortalised and validated functionally and biochemically. Validation of all knockout cell lines is included in the figures of the manuscript.

Authentication

all cell lines have been authenticated  morphologically  biochemically and functionally

Mycoplasma contamination

all cell are routinely tested every month for mycoplasma

Commonly misidentified lines
(See ICLAC register)

no misidentified cell lines were utilised

## Animals and other research organisms

Policy information about studies involving animals; ARRIVE guidelines recommended for reporting animal research, and Sex and Gender in Research

Laboratory animals

C57BL/6N

Wild animals

This study did not involve wild animals.

Reporting on sex

both males and females were used for the study. There was no difference in any of our result based on sex differences

Field-collected samples

Ad libitum access to water and standard chow was provided, while room temperature and humidity were automatically controlled. Continuous daily monitoring was conducted, and when the animals met predetermined endpoint criteria, they were humanely euthanized. All experimental procedures adhered strictly to German animal protection laws, received approval from local ethics committees, and were sanctioned by local government authorities (Landesamt für Natur, Umwelt und Verbraucherschutz Nordrhein-Westfalen).

Ethics oversight

Landesamt für Natur, Umwelt und Verbraucherschutz Nordrhein-Westfalen

Note that full information on the approval of the study protocol must also be provided in the manuscript.

## Plants

Seed stocks: n/a

Novel plant genotypes: n/a

Authentication: n/a

## Flow Cytometry

### Plots

Confirm that:

☒ The axis labels state the marker and fluorochrome used (e.g. CD4-FITC).

☒ The axis scales are clearly visible. Include numbers along axes only for bottom left plot of group (a 'group' is an analysis of identical markers).

☒ All plots are contour plots with outliers or pseudocolor plots.

☒ A numerical value for number of cells or percentage (with statistics) is provided.

### Methodology

Sample preparation: Spleen, lung and thymus were dissected from 6-8-week-old mice. Lungs were chopped and digested in 1.5 mL Liberase/DNAse digestion buffer for 30 min at 37°C. The spleens, thymi and digested lungs were passed through a 70 µM cell strainer to obtain single-cell suspensions, then centrifuged at 1,500 rpm for 5 min at 4°C. For lung and spleen, red blood cell lysis was performed using 1x RBC lysis buffer  for 4 min on ice. The reaction was stopped by addition of PBS followed by centrifugation as above. Spleens for erythrocyte analysis and thymi were not subjected to red blood cell lysis. All samples were blocked with anti-mouse CD16/32 antibody for 30 min at 4°C, then washed with FACS buffer. Cell viability was assessed by incubation with a fixable viability dye for 30 min at 4°C. After washing, cells were incubated with the respective surface antibodies in Brilliant Stain Buffer for 30 min at 4°C.

Instrument: BD FACSymphony A3 Cell Analyser

Software: Data were collected using the BD FACS Diva software. Raw FCS files were analysed using FlowJo v10.10 and statistical analysis and graphing were performed using GraphPad Prism 10.

Cell population abundance: Flow cytometry was used to assess the abundance of distinct cell populations in whole spleen, lung and thymus. Immune cells in spleen and lung were identified by CD45-BUV563 staining and further classified using the markers listed in Supplementary Table 4. Thymocytes were defined by B220-APC-R700-negativity, followed by further refinement using lineage markers. In the spleen, erythroid populations were gated as lineage-negative cells and defined by CD71-PE and TER119-BV421 expression. Cell population abundances are reported as percentages of viable cells or of the parent population, as indicated in the figure legends (Fig. 6 and Extended Data Fig. 9).

Gating strategy: For spleen, lung and thymus analysis, the starting population was defined by SSC-A/FSC-A gating and including all acquired cells. Single cells were identified via FSC-H/FSC-A and further refined by FSC-W/FSC-A gating. Viable cells were gated as APC-H7 (fixable viability dye)-negative events against SSC-A. In spleen and lung, immune cells were gated as CD45-BUV563-positive versus SSC-A and further subdivided using lineage markers listed in Supplementary Table 4. Thymocytes were gated as B220-APC-R700-negative cells versus SSC-A and further characterised by expression of CD4-BUV496 versus CD8-BV711 and CD25-BV421 versus CD44-BBB700. Erythrocyte populations in the spleen were gated as lineage-BB515-negative cells versus SSC-A, further defined by TER119-BV421 versus CD71-PE expression and subsequently clustered based on FSC-A versus CD71-PE. The full gating strategy for every organ is further specified in Supplementary Figure 2.

☒ Tick this box to confirm that a figure exemplifying the gating strategy is provided in the Supplementary Information.
